# Traditional Siddha Therapy Using Sivappu Kukkil Thailam (Topical) and Seendhil Chooranam (Oral) for Chronic Plaque Psoriasis: A Single-Patient Case Following the CAse REport (CARE) Guidelines

**DOI:** 10.7759/cureus.87732

**Published:** 2025-07-11

**Authors:** Saravanasingh Karan Chand Mohan Singh, Ethel Shiny, Jayalakshmi J, Nithyamala I, Chenthamarai Selvi G

**Affiliations:** 1 Department of Maruthuvam, National Institute of Siddha, Chennai, IND; 2 Department of Gunapadam, National Institute of Siddha, Chennai, IND; 3 Department of Sattam Sarntha Maruthuvam and Nanju Maruthuvam, Government Siddha Medical College Palayamkottai, Tirunelveli, IND

**Keywords:** dermatology life quality index, plaque psoriasis, psoriasis area severity index (pasi), seendhil chooranam, sivappu kukkil thylam

## Abstract

Psoriasis is a chronic inflammatory disease causing a significant impact on quality of life. We report a 57-year-old male with moderate-to-severe plaque psoriasis (Psoriasis Area and Severity Index (PASI) 18.6, body surface area (BSA) 22%, Dermatology Life Quality Index (DLQI) 24, pruritus 9/10), intolerant of methotrexate. Under dermatology supervision, he underwent 12 weeks of Siddha therapy (Siddha topical herbal oil (*Sivappu Kukkil Thailam *(*SKT*)) + Siddha oral powder (*Seendhil Chooranam *(*SC*))) with serial lab monitoring. Significant improvement was observed (PASI 2.4, BSA 3%, DLQI 3, pruritus 0/10), sustained at nine-month follow-up with no adverse effects. This real-world case highlights how patients can explore complementary medicine options, especially with the support of clinical supervision, and in this case, the results were quite positive. Serial photographs documented the significant resolution of plaques, although some residual post-inflammatory dyschromia remained. However, it is crucial to remember that guideline-directed therapy continues to be the cornerstone of psoriasis treatment. This uncontrolled, single-case observation does not establish Siddha therapy as a primary treatment or a valid alternative to evidence-based medicine. Instead, these findings should be viewed as preliminary data that require thorough validation. Further research, particularly randomized controlled trials that utilize standardized Siddha formulations, is essential to explore its potential role as a complementary approach within the established framework of evidence-based medicine, rather than as a replacement. Clinicians should continue prioritizing established guideline therapies while actively engaging patients in shared decision-making about complementary options.

## Introduction

Psoriasis is a chronic, immune-mediated, proliferative skin disorder. The condition is associated with red, scaly patches on the skin that can be extremely itchy. This disorder tends to be familial, and environmental triggers such as stress or infections can exacerbate the condition. The root causes are an overactive immune system, especially T cells and some cytokines, which cause skin cells to produce too rapidly and inflammation throughout the body. Tumor necrosis factor-alpha, interleukin 17 (IL-17), and interleukin 23 (IL-23) are key cytokines with important pathogenetic roles in psoriasis [1]. Psoriasis constitutes a major health burden worldwide, estimated to affect 2%-3% of the world's population. Although it predominantly affects polar regions, countries in the tropics and subtropics such as India should also be concerned about this disease. Indeed, even the prevalence of psoriasis differs substantially within India, probably because of a multitude of environmental exposures, genetic predisposition, and variations in healthcare resources [2]. Few studies in North India have reported interesting patterns of psoriasis in adults and children. It is more prevalent among adult males, who, for the most part, begin to have symptoms in their 30s or 40s [3]. In terms of gender, psoriasis affects men and women equally, although women are more likely to seek medical attention for the condition. Psoriatic arthritis, a common comorbidity, is also more prevalent in women. There is a strong familial distribution in the predisposition to psoriasis. There is evidence from studies which suggests that patients who have a positive psoriasis family history are first-degree relatives with psoriasis and thus bring them closer to the psoriasis disease, the former represents approximately 40% of patients with this disease. Patients with family history also tend to get diseases at a younger age and with more severe symptoms, such as psoriatic arthritis and nail changes [4,5]. Current management of psoriasis relies on systemic and biologic therapies, which carry a recognized spectrum of potential adverse effects. Established systemic agents, including methotrexate, cyclosporine, and acitretin, demonstrate efficacy but are associated with significant risks requiring vigilant monitoring. Documented adverse effects include hepatotoxicity, nephrotoxicity, hypertension, teratogenicity, and an increased risk of malignancy [6-8]. These safety profiles have stimulated research interest in complementary therapeutic approaches, including traditional medicine systems such as Siddha and Ayurveda. This case report documents the adjunctive use of Siddha medicine in the management of psoriasis, observing a favorable clinical response. The patient was treated with application of a Siddha topical herbal oil (*Sivappu Kukkil Thailam* (*SKT*)) and a Siddha oral powder (*Seendhil Chooranam* (*SC*)). During the documented follow-up period, the patient experienced resolution of psoriatic lesions with no observed recurrence while maintained on the Siddha therapy regimen. This observation does not establish Siddha therapy as a primary or alternative treatment. Evidence-based guideline therapy remains the standard of care for psoriasis; any complementary approaches, including Siddha, must be investigated rigorously within this framework before claims of efficacy can be made.

## Case presentation

A 57-year-old Tamil farmer presented with a six-year history of gradually progressive plaque psoriasis that had become resistant to high-potency topical corticosteroids. He also experienced significant intolerance to methotrexate (15 mg/week), which he had to stop after 24 weeks due to severe nausea. He reported severe pruritus, embarrassment at visible scale, and difficulty working outdoors; the Dermatology Life Quality Index (DLQI) was 24/30, signifying a “very large” impact on daily life. Past medical history was notable only for well-controlled essential hypertension; there was no personal or family history of psoriatic arthritis, metabolic syndrome, or autoimmune disease. Examination revealed multiple, well-demarcated, erythematous plaques with thick micaceous scale over the trunk, lumbosacral region, scalp, elbows, knees, and extensor forearms, involving approximately 22% of body surface area. Joints were nontender and nonswollen. The Psoriasis Area and Severity Index (PASI) was 18.6, consistent with moderate-to-severe disease [9]. Vital signs were stable; body mass index was 28 kg m⁻². Baseline laboratory screening, including complete blood count, liver and renal panels, fasting glucose and lipids, hepatitis B/C serology, and HIV test, was within normal limits. The clinical morphology was deemed classic, and the patient declined skin biopsy. After shared decision-making that emphasized the adjunctive nature of traditional remedies and the option to resume evidence-based systemic therapy at any time, the patient consented to a 12-week trial of Siddha medicine and routine laboratory monitoring every four weeks.

Diagnosis

The lesion was clinically diagnosed as plaque psoriasis by a dermatologist. Both general and systemic examinations yielded no remarkable findings, and no other abnormalities were noted during the evaluation.

Therapeutic intervention

The treatment protocol involved obtaining written informed consent and included two Siddha formulations. First, the formulation of *SKT* begins with a decoction of crushed *Rubia cordifolia *and* Hemidesmus indicus* roots in eight parts water, boiled down to one-eighth; the filtrate is combined with an equal volume of gingelly (sesame) oil and reheated. A paste (karkam) of the same two herbs is incorporated, after which beeswax and finely pulverized white *Kungiliyam* (resin of *Vateria indica*) are added to improve viscosity and create an occlusive film. The mixture is gently sand-bath heated, filtered, and finally enriched with powder of *Vempadam Pattai* (bark of *Alkanna tinctoria*) [10]. *SKT* was applied as a thin film to every psoriatic plaque twice daily after a lukewarm bath.

Second, the patient ingested *SC*. The preparation begins by rinsing *Seenthil* (*Tinospora cordifolia*) stems 21 times with clean water, shade-drying them, macerating the stems in cow’s milk, and drying again to complete moisture removal. An equal weight of finely powdered* Karisalai leaf* (*Eclipta prostrata*) is then blended with the milk-treated *Seenthil*. Next, live *Poonagam* (earthworms, *Eudrilus eugeniae*) are soaked in milk to expel soil, sun-dried, pulverized, and washed three additional times in fresh milk. After filtration and thorough drying, equal weight of this *Poonagam* powder is incorporated into the *Seenthil-Karisalai* mixture, and the composite is triturated to a uniform, moisture-free chooranam [11]. The recommended dosage was 2 g taken twice daily with ghee after meals. No topical corticosteroids, phototherapy, or additional systemic agents were permitted during the 12-week observation.

Follow-up and outcomes

**Figure 1:**
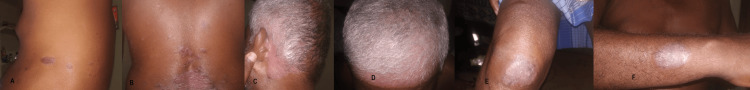
Baseline cutaneous findings before initiation of therapy A. Lateral trunk (flank): discrete psoriatic plaques coalescing into larger confluent lesions, showing typical peripheral erythema and central scaling. B. Lumbosacral region: well-demarcated erythematous plaques with thick, micaceous (silvery-white) scale. C. Frontal scalp/hairline: psoriatic scalp involvement with adherent silvery scale extending 1-2 cm beyond the hairline. D. Post-auricular folds: erythematous plaques with marked scaling in the retro-auricular crease. Mild fissuring present. E. Right knee joint: classic thick hyperkeratotic plaques with accentuated silvery scale. F. Dorsal forearm: coin-shaped psoriatic plaques.

**Figure 2:**
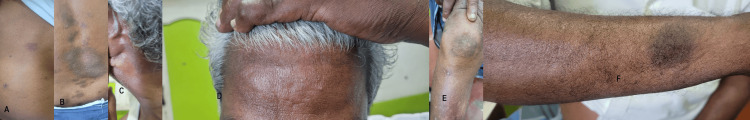
Photographs illustrating the patient's improvement post-treatment A. Lateral trunk: shows post-inflammatory hyperpigmentation. B. Lumbosacral region: marked reduction in lesions, with re-epithelialization and post-inflammatory hyperpigmentation. C. Post-auricular fold: only scant residual papules remained, indicating substantial clinical recovery. D. Scalp: smoother surface and no scaling. E. Right knee joint: the skin appears smooth and nearly plaque-free. F. Forearm: complete plaque resolution and post-inflammatory hyperpigmentation.

Over a 12-week observational period, the patient showed impressive improvements with the Siddha regimen, which involved *SKT* and *SC*. His PASI score dropped significantly from 18.6 to 2.4. Additionally, body surface involvement decreased dramatically from 22% to 3%. The level of pruritus, which had previously been a troublesome 9 out of 10, now stood at 0 out of 10. In terms of quality of life, the DLQI saw a remarkable improvement, going from 24, indicating a "very large" impact to 3, which reflects a "small" impact. Photographs taken throughout this period clearly displayed notable flattening of plaques and a reduction in micaceous scale, as shown in Figures 1A-1F, 2A-2F. This progress allowed the patient to return to work without feeling embarrassed. Laboratory monitoring remained stable, and no heavy metals were found in the plasma. The patient maintained an adherence rate of over 95% to the treatment regimen. During a shared-decision visit, he chose to continue with the current treatment plan, opting for quarterly reviews and using topical calcipotriol-betamethasone as a rescue treatment. The strategy includes the option to escalate to conventional systemic or biologic therapy only if there is a decline in PASI or DLQI. During the follow-ups at six and nine months, everything looked great. There was no sign of recurrence or adverse effects. The patient remained completely symptom-free, and there were no complications to report.

## Discussion

This case demonstrates a remarkably successful 12-week trial of Siddha medicine *SKT* and *SC* in a patient with moderate-to-severe plaque psoriasis and achieving near-complete clearance (PASI 18.6 to 2.4) and a dramatic improvement in quality of life (DLQI 24 to 3) [12].

Plausibility mechanism 

*SKT* combines six ingredients whose phytochemicals act on complementary nodes of psoriatic inflammation. Anthraquinones (alizarin, purpurin) from *Rubia cordifolia* suppress nuclear factor kappa-light-chain-enhancer of activated B cells (NF-κB)/mitogen-activated protein kinase (MAPK) signaling and lower tumor necrosis factor alpha (TNF-α), interleukin 6 (IL-6), and IL-17 output in activated macrophages [13]. The ethyl acetate fraction of *Rubia cordifolia* has been shown to inhibit keratinocyte proliferation and promote differentiation, suggesting its potential as an antipsoriatic agent [14]. *Hemidesmus indicus* has been noted for its anti-inflammatory properties, which are crucial in managing conditions such as psoriasis where keratinocyte proliferation is dysregulated [15]. Resin of *Vateria indica* containing bergenin was shown to reduce inflammation by downregulating the phosphorylation of NF-κB and MAPK signaling pathways, leading to decreased levels of pro-inflammatory cytokines such as nitric oxide (NO), TNF-α, interleukin-1 beta (IL-1β), and IL-6 [16]. Sesame oil itself is rich in sesamin and other lignans, which have been shown to interact efficiently with cyclooxygenase-2 (COX-2), further supporting its anti-inflammatory potential [17]. The use of beeswax in topical formulations enhances skin barrier function by decreasing transepidermal water loss and increasing stratum corneum water content, which aids in the occlusion and hydration of the skin [18]. The study concludes that the herbal extracts from *Tinospora cordifolia* exhibit significant potential in alleviating the symptoms and underlying processes of psoriasis, like dermatitis in mice, primarily due to their anti-inflammatory properties that counteract pro-inflammatory mediators associated with the disease [19]. An α-d-glucan, exhibiting unique immune-stimulating properties, is isolated and characterized from the medicinal plant *Tinospora cordifolia *[20]. *Eclipta prostrata* coumestans (wedelolactone) shows activity against inflammatory and tumor-promoting events in murine skin, depicting plausible role of NF-kB pathway [21]. Lumbrokinase, a fibrinolytic enzyme derived from earthworms, exhibits strong fibrinolytic activity by breaking down fibrin and plasminogen, which can enhance microcirculation in affected skin areas, potentially alleviating symptoms associated with psoriatic lesions [22].

Limitations

The study design relies on a single patient and is uncontrolled, which means we cannot rule out factors such as regression to the mean, seasonal variations, the Hawthorne effect, or even increased exposure to ultraviolet light that comes with farming. Additionally, since there was no histopathological examination, the diagnosis depended on classic morphology, leaving the possibility that other papulosquamous disorders were not definitively excluded. This study does have its limitations, primarily because it is based on just one case report. This underscores the need for more systematic clinical studies with larger populations to validate the effectiveness of the remedy used. While this case offers some valuable insights, it does not provide robust evidence. It is vulnerable to biases and heuristics that can affect clinical practice and influence scientific literature. Moreover, this type of study does not generate epidemiological data, making it difficult to draw broader conclusions or comprehensive insights regarding the management of psoriasis in various contexts. Therefore, we cannot arrive at definitive conclusions about treatment and management for this condition in future cases based solely on a single case report.

## Conclusions

This single-case report indicates that the Siddha preparations *Sivappu Kukkil Thailam* and *Seendhil Chooranam*, when used under the guidance of a dermatologist, led to significant clinical improvement in a patient with chronic plaque psoriasis. However, it is important to note that this observation lacks a controlled and blinded design and is limited to just one patient over a nine-month period. Therefore, it cannot definitively establish the efficacy, safety, or general applicability of these treatments. Currently, the gold standard for treating psoriasis includes guideline-directed topical, systemic, and biologic therapies that have been validated through randomized controlled trials. Traditional Siddha remedies should be viewed as investigational adjuncts, utilized only through informed shared decision-making and careful monitoring. To recommend any complementary therapy beyond generating hypotheses, robust multi-center, placebo-controlled studies using standardized preparations are essential.
